# Exosomes derived from stem cells from apical papilla promote craniofacial soft tissue regeneration by enhancing Cdc42-mediated vascularization

**DOI:** 10.1186/s13287-021-02151-w

**Published:** 2021-01-22

**Authors:** Yao Liu, Xueying Zhuang, Si Yu, Ning Yang, Jianhong Zeng, Xuemei Liu, Xu Chen

**Affiliations:** 1 Department of Pediatric Dentistry, School and Hospital of Stomatology, China Medical University, 117 Nanjing North Street, Shenyang, 110002 China; 2 Liaoning Provincial Key Laboratory of Oral Diseases, Shenyang, China

**Keywords:** Stem cells from apical papilla, Exosome, Vascularization, Angiogenesis, Tissue regeneration, Cell migration, Cell division cycle 42

## Abstract

**Background:**

Reconstruction of complex critical-size defects (CSD) in the craniofacial region is a major challenge, and soft tissue regeneration is crucial in determining the therapeutic outcomes of craniofacial CSD. Stem cells from apical papilla (SCAP) are neural crest-derived mesenchymal stem cells (MSCs) that are homologous to cells in craniofacial tissue and represent a promising source for craniofacial tissue regeneration. Exosomes, which contain compound bioactive compounds, are the key factors in stem cell paracrine action. However, the roles of exosomes derived from SCAP (SCAP-Exo) in tissue regeneration are not fully understood. Here, we explored the effects and underlying mechanisms of SCAP-Exo on CSD in maxillofacial soft tissue.

**Methods:**

SCAP-Exo were isolated and identified by transmission electron microscopy and nanoparticle tracking analysis. The effects of SCAP-Exo on wound healing and vascularization were detected by measuring the wound area and performing histological and immunofluorescence analysis on the palatal gingival CSD of mice. Real-time live-cell imaging and functional assays were used to assess the effects of SCAP-Exo on the biological functions of endothelial cells (ECs). Furthermore, the molecular mechanisms of SCAP-Exo-mediated EC angiogenesis in vitro were tested by immunofluorescence staining, Western blot, and pull-down assays. Finally, in vivo experiments were carried out to verify whether SCAP-Exo could affect vascularization and wound healing through cell division cycle 42 (Cdc42).

**Results:**

We found that SCAP-Exo promoted tissue regeneration of palatal gingival CSD by enhancing vascularization in the early phase in vivo and that SCAP-Exo improved the angiogenic capacity of ECs in vitro. Mechanistically, SCAP-Exo elevated cell migration by improving cytoskeletal reorganization of ECs via Cdc42 signalling. Furthermore, we revealed that SCAP-Exo transferred Cdc42 into the cytoplasm of ECs and that the Cdc42 protein could be reused directly by recipient ECs, which resulted in the activation of Cdc42-dependent filopodium formation and elevation in cell migration of ECs.

**Conclusion:**

This study demonstrated that SCAP-Exo had a superior effect on angiogenesis and effectively promoted craniofacial soft tissue regeneration. These data provide a new option for SCAP-Exo to be used in a cell-free approach to optimize tissue regeneration in the clinic.

**Supplementary Information:**

The online version contains supplementary material available at 10.1186/s13287-021-02151-w.

## Background

Complex critical-size defects (CSD) in the craniofacial region caused by trauma, tumours, infection, and maxillofacial surgery severely affect the physical appearance of patients and the function of the oral cavity, posing major challenges for reconstructive surgery in the clinic [1–3]. The reconstruction of CSD in the craniofacial region combines regeneration of a variety of tissues, including bone, muscle, mucosal tissues, and skin. Although bone regeneration guides the structural stability and appearance of the face, the overlying soft tissue is essential for bone regeneration and for restoration of orofacial function and aesthetic integrity [4]. Advances in tissue engineering have demonstrated that the successful regeneration of soft tissues helps to prevent exogenous infection, provide sufficient nutrients, and establish blood supply, as well as providing other benefits [5]. Moreover, a large number of clinical studies have identified that natural free flaps can be used to effectively achieve soft tissue regeneration, significantly improving the outcomes of craniofacial reconstruction [6]. It is well known that the vascular system is required for embryonic development and tissue regeneration, and adequate blood vessel formation enables supply of sufficient oxygen and nutrients and elimination of metabolic waste [7]. Therefore, vascularization in the early phase of wound healing plays a critical role in the regeneration of craniofacial soft tissue [8]. A strategy based on bioactive factors derived from mesenchymal stem cells (MSCs) has been proposed as a promising approach for regenerative medicine [9]. Exosomes, some of the most important extracellular microvesicles secreted from stem cells, contain many cytoplasmic components, such as proteins, peptides, RNA, and DNA [10]. Increasing evidence has demonstrated that exosomes have functions similar to those of donor MSCs, which regulate signal transcription and protein expression to mediate the cellular functions of recipient cells [11]. In the process of tissue regeneration, exogenous MSCs localize around endothelial cells (ECs) and promote angiogenesis by secreting exosomes [12, 13]. Notably, compared to MSCs, exosomes may have a superior safety profile and can be stably stored without losing their cellular functions, which successfully overcomes some of the major challenges related to the cell-based approach of regenerative medicine.

Stem cells from apical papilla (SCAP), which are a type of neural crest-derived MSCs, are homologous to cells of the craniofacial region and have been identified as a promising stem cell source for tissue engineering with high self-renewal and multilineage differentiation capacities [14]. Since they are isolated from the highly vascularized developing apical tissue of immature permanent teeth, SCAP may have excellent properties for the promotion of angiogenesis [15]. Even under conditions of microenvironment stress with oxygen, serum and glucose deficiency, SCAP maintain their biological activity and secrete large amounts of pro-angiogenic growth factors while secreting reduced amounts of anti-angiogenenic growth factors to promote EC angiogenesis [16]. In addition, SCAP enhance vascularization to improve pulp-like tissue regeneration in vivo, emphasizing their promising roles in tissue engineering [17]. Angiogenesis is a complex and highly precise process that includes endothelial progenitor cell activation, EC proliferation, migration, sprouting, and neovascularization [18]. Among these processes, the migration of ECs mediated by cytoskeletal reorganization might play an important role in angiogenesis. Rho GTPase family members, including cell division cycle 42 (Cdc42), Rac1, and RhoA, are important molecular switches that regulate cytoskeletal reorganization [19]. Therefore, whether exosomes derived from SCAP (SCAP-Exo) promote angiogenesis and soft tissue regeneration and the possible mechanisms of such effects were the main foci of this study.

In this study, we explored the effects and potential application of SCAP-Exo for craniofacial soft tissue regeneration in vivo*.* Our findings suggested that SCAP-Exo increased EC migration and angiogenic capacity via Cdc42-dependent cytoskeletal reorganization, which resulted in the promotion of tissue regeneration of palatal gingival CSD. To the best of our knowledge, this is the first study indicating that SCAP-Exo can be used in a cell-free approach to optimize soft tissue regeneration in the clinic.

## Materials and methods

### Animals

C57BL/6J mice and BALB/c nude mice were purchased from Vital River Laboratory Animals Technology (Beijing, China). All animal experiment protocols (2018029) were approved by the Institutional Animal Care and Use Committee of China Medical University.

### Antibodies and reagents

Anti-CD9, anti-CD63, anti-Alix, anti-calnexin, anti-CD31, anti-CD34, anti-CD45, anti-CD73, anti-CD90, anti-CD105, anti-Collagen I, and anti-Fibronectin antibodies were purchased from Abcam (Cambridge, UK). Anti-Ki-67, anti-RhoA, anti-Rac1, anti-Cdc42, and anti-β-actin antibodies were purchased from Cell Signaling Technology (Danvers, USA). Alexa Fluor 488- and Alexa Fluor 568-conjugated secondary antibodies were purchased from Proteintech (Rosemont, IL, USA). IRDye 800cw-conjugated goat anti-rabbit/anti-mouse IgG secondary antibodies were purchased from Abbkine (Redlands, CA, USA). PKH-26 and PKH-67 kits were purchased from Sigma-Aldrich (St. Louis, MO, USA). Lipofectamine™ RNAiMAX, FM™ 4-64FX, and ActinGreen™ 488 were purchased from Thermo Fisher (Eugene, Oregon, USA). SiCdc42, Cdc42-EGFP, and Cdc42-mCherry fusion protein expression plasmids were purchased from GenePharma (Suzhou, China). The Cdc42 inhibitor ML141 was purchased from MedChemExpress (Monmouth Junction, USA).

### SCAP isolation and characterization

Human third molars with immature roots were obtained from healthy donors aged 12 to 15 years in the clinic at the School of Stomatology affiliated with China Medical University. Informed consent was obtained from all patients and their parents. The apical papilla was gently separated and digested with dispase II (Boehringer Ingelheim, Mannheim, Germany) and collagenase type I (Worthington Biochemical Co., Lakewood, CO, USA). Single-cell suspensions were seeded and cultured in alpha-minimum essential medium (α-MEM, HyClone, Logan, UT, USA) supplemented with 15% (v/v) foetal bovine serum (FBS, MRC, Uruguay), 1% (v/v) penicillin-streptomycin solution (HyClone), 2 mM L-glutamine (BioSource/Invitrogen, USA), and 0.1 mM L-ascorbic acid (Sigma-Aldrich, St. Louis, MO, USA) and incubated at 37 °C with 5% CO_2_. The expression of MSC surface markers, including CD31, CD34, CD45, CD73, CD90, and CD105, was detected by flow cytometry. The multipotent differentiation potential of SCAP, including the potential for osteogenesis and adipogenesis, was evaluated using osteogenic and adipogenic differentiation media for 4 weeks. Alizarin red S and oil red O staining were used to detect the formation of mineralized nodules and lipid droplets, respectively.

### SCAP-Exo isolation and identification

SCAP were cultured in exosome-free medium for 48 h. The culture supernatant was collected and centrifuged at 4 °C in an ultracentrifuge at three different speeds: 3000×*g* for 20 min, 20,000×*g* for 30 min, and 120,000×*g* for 2 h (Beckman Optima L-100XP, USA). The exosomes were resuspended in sterile PBS and stored at − 80 °C. SCAP-Exo were observed by transmission electron microscopy (TEM) (H-800, Hitachi, Japan). A nanoparticle tracer analyser (ZetaView, Germany) was used to measure the sizes of the particles. Exosomal surface markers, including CD9, CD63, and Alix, were detected by Western blotting. Cdc42 siRNA and the Cdc42 inhibitor ML141 (20 μM) were used to treat SCAP (whole cells). The exosome-free medium was changed and the cells were cultured for 48 h. The culture supernatant was collected and centrifuged to isolate SCAP^siCdc42^-Exo and SCAP^ML141^-Exo.

### Quantification of exosomes

Twenty microlitres of SCAP-Exo were added to 50 μL of protein lysate and lysed on ice for 1 h. A BCA protein assay kit was used to generate a standard curve of the protein concentration and was subsequently used to measure the concentration of SCAP-Exo.

### SCAP-Exo treatment in wound healing of CSD in the palatal gingiva

The wound model was identical to that in a previous study [20]. Full-thickness circular gingival wounds (soft tissue defects) with a diameter of 2.0 mm were made in the palates of C57BL/6J mice using a biopsy punch (*n* = 5). SCAP-Exo or SCAP^siCdc42^-Exo was suspended in PBS at a concentration of 1 μg/μL. Forty microlitres SCAP-Exo, SCAP^siCdc42^-Exo, or PBS (as control) was injected submucosally into four symmetrical sites around the wounds after they were created, according to a previous protocol [21], so that 40 μg of exosomes were applied locally on each wound. The mice were sacrificed 7 days post-operation. Tissue samples were fixed in 4% paraformaldehyde and decalcified with 10% ethylenediaminetetraacetic acid solution. The samples were embedded in paraffin, sectioned, and stained with haematoxylin and eosin (H&E). In addition, the samples were embedded in optimal cutting temperature compound and sectioned. In order to characterize the newly healed gingival tissues, the frozen sections were stained with immunofluorescent Collagen I and Fibronectin which are the matrix markers. The frozen sections were also stained with immunofluorescent CD31, an endothelial marker of micro-vessels, and the CD31-positive area was analysed using the ImageJ software (1.50i, National Institutes of Health, Bethesda, MD, USA).

### In vivo tracking experiment

PKH-26-labelled SCAP-Exo or PBS (as a control) was injected submucosally into four symmetrical sites around the wounds once after the wounds were created. The mice were sacrificed 7 days post-operation. Frozen sections and fluorescence images were used to observe the fates of SCAP-Exo.

### Real-time live-cell imaging (RT-LCI)

Human umbilical vein endothelial cells (HUVECs) (American Type Culture Collection, Rockvile, MD, USA) were seeded into a 35-mm dish (81,158, Ibidi, Germany) and stained with FM™ 4-64FX. SCAP-Exo were labelled with PKH-67 and added to the HUVECs. The process of SCAP-Exo uptake by HUVECs was observed under a laser confocal microscope (ECLIPSE Ti2, Nikon, Japan) for 30 min continuously.

### Western blot analysis

Protein concentrations were detected using a BCA protein assay kit. Proteins (20 μg) were loaded onto a 12% sodium dodecyl sulphate-polyacrylamide gels for electrophoresis and then transferred to polyvinylidene difluoride membranes. The membranes were exposed to the appropriate primary and secondary antibodies. Finally, the bands were revealed using an Odyssey CLx instrument (LI-COR, Lincoln, NE, USA). The density of each band was measured with ImageJ to quantify protein expression.

### Tube formation assay

Matrigel (50 μL) (#356234, BD Biosciences, San Jose, CA) was used to precoat each well of a 96-well plate and polymerized at 37 °C. HUVECs and SCAP-Exo-pretreated HUVECs were seeded at a density of 1.5 × 10^4^ cells/well and cultured for 8 h. Photographs of tube formation were taken with a stereoscopic microscope (ECLIPSE TE2000-S, Nikon, Japan). The indexes of tube formation were analysed using ImageJ.

### Matrigel plug assay

Matrigel (200 μL) (#356231, BD Biosciences, San Jose, CA) was mixed with SCAP-Exo, SCAP^siCdc42^-Exo, SCAP^ML141^-Exo, or PBS on ice. The mixtures were injected subcutaneously into the backs of BALB/c nude mice *(n* = 5). After 14 days, the Matrigel plugs were extracted. H&E staining was used and the vessels in the Matrigel were counted.

### Cell proliferation assay

Cell proliferation was measured using cell counting kit-8 (CCK-8) and Ki-67 staining assays. HUVECs were seeded into 96-well plates at a density of 2000 cells/well and cultured with SCAP-Exo. The plates were incubated for 24, 48, and 72 h. CCK-8 solution (Dojindo, Kumamoto, Japan) was added, and the plates were incubated in the dark. The absorbance of each well was measured at 450 nm using a microplate reader (Tecan, Salzburg, Austria). In addition, HUVECs (2 × 10^4^/well) were seeded on glass coverslips placed inside a 12-well plate and cultured to the logarithmic phase. Thereafter, the cells were fixed and stained with an immunofluorescent Ki-67 antibody. The number of Ki-67-positive cells was indicated as a percentage of the total cell number.

### Cell migration assay

Cell migration was measured using transwell cell migration and scratch wound healing assays. HUVECs were seeded into the upper Transwell insert of a 24-well plate at a density of 1 × 10^4^ cells/well. SCAP-Exo were added to the lower chamber and incubated for 24 h. Thereafter, the cells in the Transwell chamber were removed. After staining with crystal violet, the cells that migrated below the Transwell layer were counted. Moreover, HUVECs (5 × 10^5^/well) were seeded into a 6-well plate, and a scratch in the cells was made with a 200-μL sterile tip. The medium was then replaced with serum-free medium containing SCAP-Exo. After 0, 12, and 24 h, the boundaries of the scratches were recorded and the wound closure rates were calculated using ImageJ.

### Pull-down assay

A RhoA/Rac1/Cdc42 Activation Assay Combo Biochem Kit (Cytoskeleton, Denver, CO, USA) was used following the manufacturer’s instructions. Briefly, equivalent protein amounts of lysate were added to a pre-determined amount of rhotekin-RBD (for RhoA activation) or PAK-PBD beads (for Rac1 and Cdc42 activation) and incubated at 4 °C on a rotator for 1 h. Next, the beads were centrifuged and washed. The bead pellets were resuspended in 20 μL of loading buffer and boiled. The samples were then analysed by Western blot assay.

### F-actin immunofluorescence staining

HUVECs were fixed for 30 min and stained with ActinGreen™ 488 at 4 °C for 30 min. Pseudopodia formation was observed by fluorescence microscopy (ECLIPSE 80i, Nikon, Japan). We counted the filopodia and used ImageJ software to quantitatively analyse the lengths of filopodia.

### Plasmid transfection and fluorescence co-localization

Cdc42-EGFP or Cdc42-mCherry fusion protein expression plasmids were transfected into SCAP, and SCAP^Cdc42-EGFP^-Exo or SCAP^Cdc42-mCherry^-Exo was extracted. SCAP^Cdc42-EGFP^-Exo was added to HUVECs, and the cells were passaged to the 6th passage. SCAP^Cdc42-mCherry^-Exo was added to HUVECs, and the cells were incubated overnight. The cells were then incubated with Cdc42 primary antibody and fluorescent secondary antibody. The co-localization of Cdc42 and Cdc42-mCherry was observed by confocal microscopy (ECLIPSE Ti2, Nikon).

### Statistical analysis

All data were recorded as the mean ± standard deviation (SD). Comparisons between two groups were analysed using an independent two-tailed Student’s *t* test, and comparisons among more than two groups were performed using one-way analysis of variance (ANOVA) with SPSS 20.0 (SPSS Inc., Chicago, IL, USA). A value of *P* < 0.05 was considered to indicate statistically significance.

## Results

### Characterization of SCAP and identification of SCAP-Exo

The SCAP were spindle-shaped cells in primary culture (Fig. S1a). Under in vitro osteogenic and adipogenic induction conditions, SCAP formed mineralized nodes and oil droplets, as assessed by Alizarin red S staining and oil red O staining (Fig. S1b-c). Flow cytometry analysis showed that SCAP expressed MSC surface markers, including CD73, CD90, and CD105, while the haematopoietic markers CD31, CD34, and CD45 were absent (Fig. S1d).

We isolated extracellular vesicles (EVs) from the culture supernatant of SCAP. The EVs exhibited bilayer membranes and cup-shaped structures under TEM (Fig. S2a). Nanoparticle tracking analysis showed that EVs with diameters of approximately 120.1 nm accounted for 97.2% of nanoparticles, and the mean diameter of EVs was 139.2 ± 62.5 nm (Fig. S2b). Western blot analysis showed that EVs expressed the exosomal markers Alix, CD9, and CD63 but failed to express calnexin (Fig. S2c). Therefore, following the guidelines for the minimal information from studies on EVs [22], we identified the EVs isolated from SCAP as exosomes.

### SCAP-Exo promoted vascularization to accelerate tissue regeneration of the palatal gingiva

**Fig. 1:**
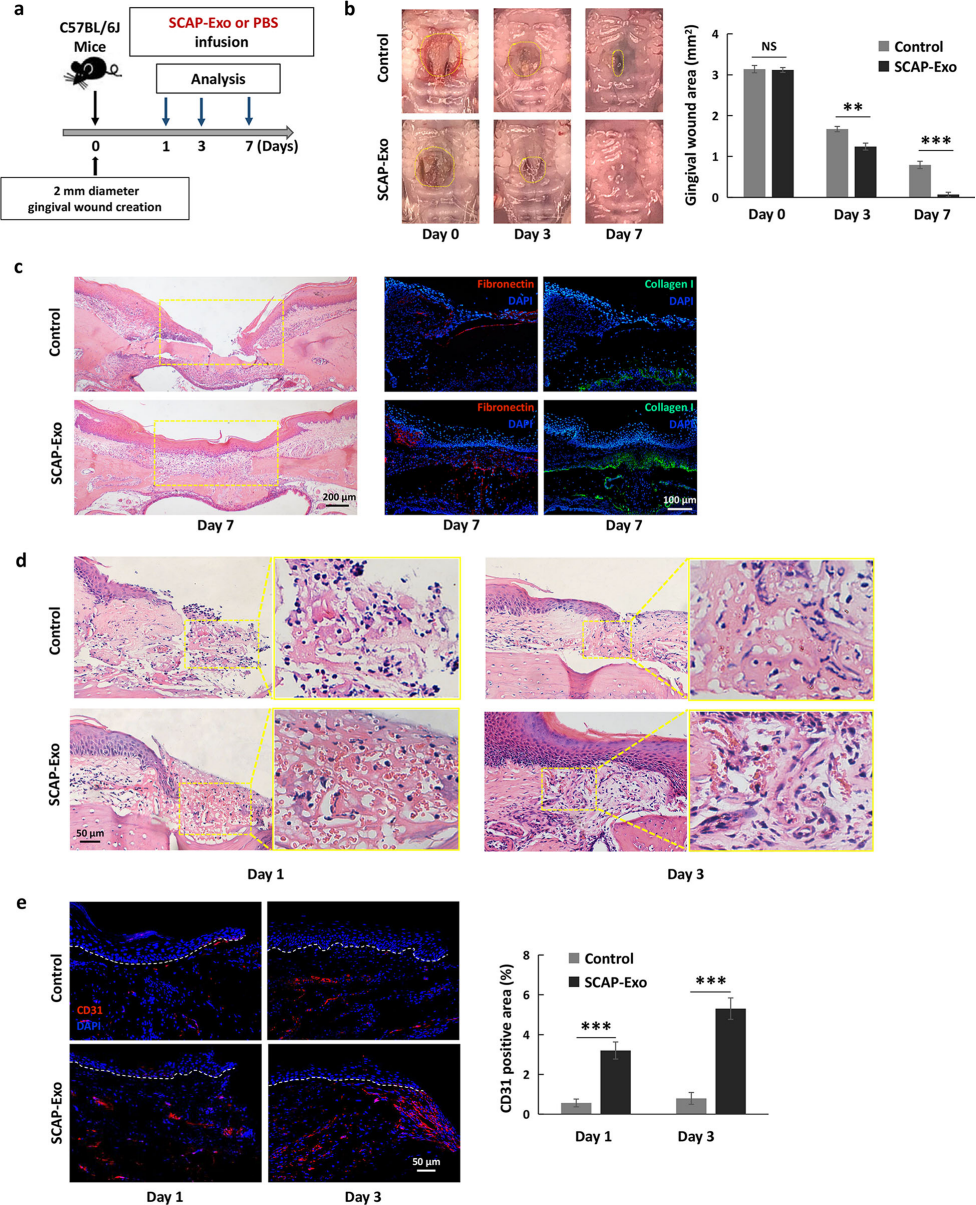
SCAP-Exo promoted angiogenesis to accelerate wound healing in the palatal gingiva. **a** Schematic indicating the experimental design for SCAP-Exo infusion in gingival wounds in the palates of mice. **b** Representative images and quantification of the gingival wound areas in the control and SCAP-Exo groups. Wound healing of the palatal gingiva was significantly accelerated in the SCAP-Exo group at 3 and 7 days post wounding compared to the control group. **c**. H&E staining was used to observe the epidermis and connective tissues formation at 7 days post wounding. Scale bar = 200 μm. Immunofluorescence staining showing significantly increased Collagen I and Fibronectin expressions in the palatal gingiva of the SCAP-Exo group at 7 days post wounding compared with the control group. Scale bar = 100 μm. **d** Histological views showing many newly formed blood vessels containing red blood cells in the gingival wounds of the SCAP-Exo group at 1 and 3 days post wounding compared with the control group. Scale bar = 50 μm. **e** Immunofluorescence staining and quantification showed that the percentage of CD31-positive area (red) in the SCAP-Exo group was higher than that in the control group at 1 and 3 days post wounding. The epidermis and connective tissues are separated by the white dotted line in the images. The slides were counterstained with DAPI (blue). Scale bar = 50 μm. *n* = 5 in each group. NS: *P* > 0.05. **P < 0.01, ***P < 0.001. Error bars: mean ± SD

We locally infused SCAP-Exo into CSD in the palatal gingiva in mice and analysed the therapeutic effects at 1, 3, and 7 days (Fig. 1a). The in vivo tracking experiment showed that PKH-26-labelled SCAP-Exo could exist in palatal gingival defects until 7 days post-operation (Fig. S3). We found that the wound area of the palatal gingiva was significantly smaller in the SCAP-Exo infusion group at 3 and 7 days post wounding than in the control group (Fig. 1b). Newly formed and integral epidermis and connective tissues were observed in the SCAP-Exo group using H&E staining at 7 days post wounding, while the formation of epidermis and connective tissues was markedly delayed in the control group. Immunofluorescence staining showed that the expression levels of Collagen I and Fibronectin in palatal gingiva defects in the SCAP-Exo infusion group at 7 days post wounding were significantly higher than in the control group, which demonstrated more new formation of gingival tissue in SCAP-Exo group (Fig. 1c). We further focused on vascularization in the early phase of healing of the palatal gingival CSD. H&E staining showed that there were significantly more newly formed blood vessels in the gingival wounds in the SCAP-Exo group than in the control group at 1 and 3 days post wounding (Fig. 1d). Immunostaining confirmed that the percentage of the CD31-positive area was significantly increased in the SCAP-Exo group at 1 and 3 days post wounding (Fig. 1e). Therefore, our data indicated that SCAP-Exo promoted vascularization in the early phase of healing and accelerated tissue regeneration of the palatal gingiva.

### SCAP-Exo improved the angiogenic capacity of HUVECs

To determine the effects of SCAP-Exo on angiogenesis, we used SCAP-Exo to treat HUVECs in vitro*.* RT-LCI showed that SCAP-Exo were endocytosed into the cytoplasm of HUVECs (Fig. S4a-e). Based on analysis of the *X*-*T*-, *Y*-*T*-, and *X*-*Y*-*Z*-axis images, we observed the entire process of SCAP-Exo uptake into HUVECs (Fig. S4f-h). To select an optimal concentration of SCAP-Exo, different doses (5–20 μg/mL) of SCAP-Exo were added to HUVECs. We found that SCAP-Exo increased the expression of the angiogenic protein CD31 in HUVECs in a dose-dependent manner (Fig. S5). Therefore, we used 20 μg/mL SCAP-Exo in subsequent experiments.

**Fig. 2:**
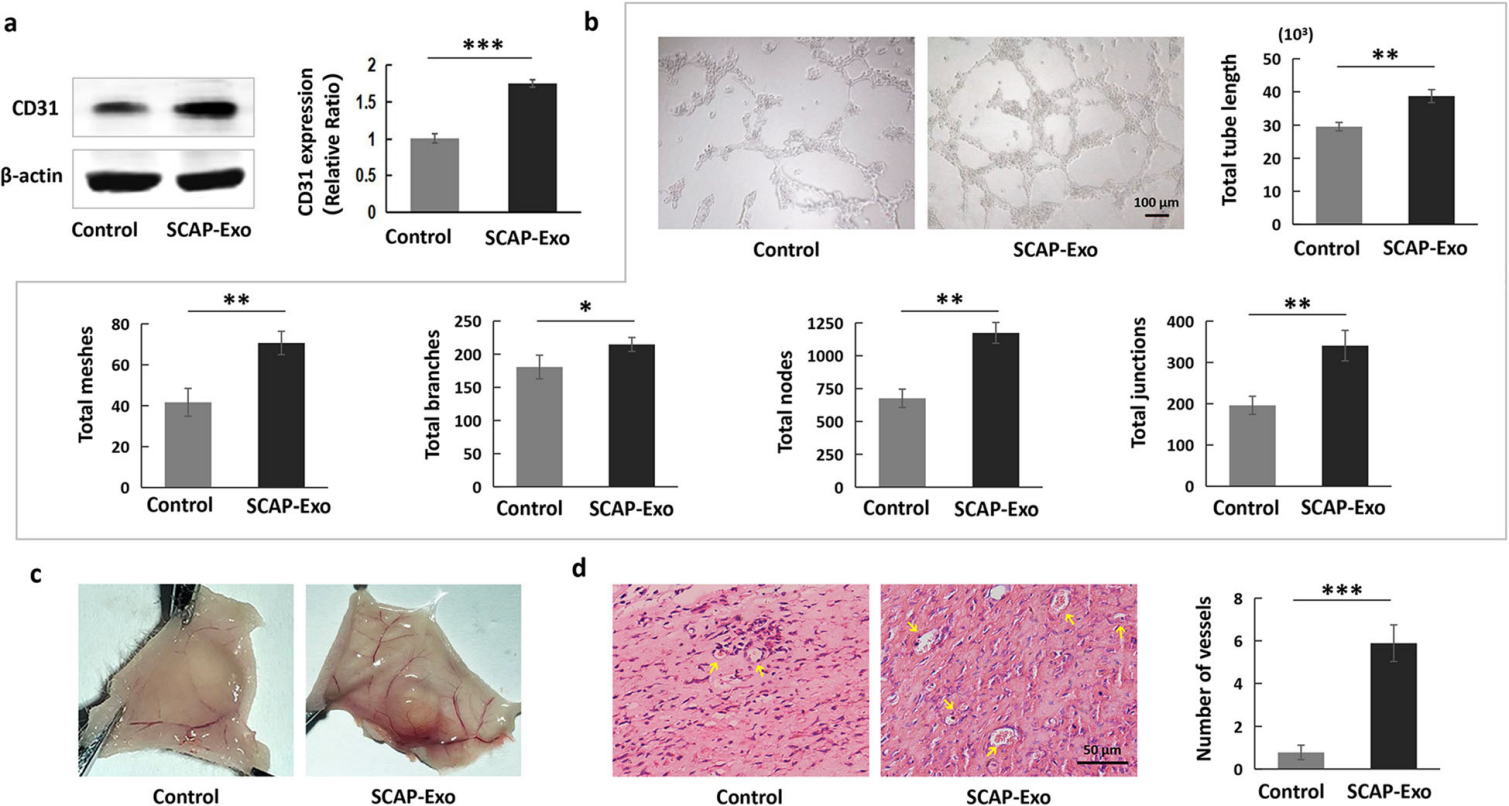
SCAP-Exo improved the angiogenic capacity of HUVECs. **a** Western blot analysis showed that SCAP-Exo treatment upregulated the expression levels of CD31 in HUVECs. **b** The in vitro tube formation assay showed that the non-treated HUVECs were scattered and formed relatively few lumens, while SCAP-Exo-treated HUVECs formed complete lumens. The levels of variables including total tube length and total numbers of meshes, branches, nodes, and junctions were higher in SCAP-Exo-treated HUVECs than in non-treated HUVECs. Scale bar = 100 μm. **c** Representative images of Matrigel plugs showing that there were more newly formed blood vessels in the SCAP-Exo group than in the control group. **d** H&E staining showing that there were more vascular lumens (yellow arrow) containing red blood cells in the SCAP-Exo group than in the control group. Scale bar = 50 μm. *n* = 5 in each group. *P < 0.05, **P < 0.01, ***P < 0.001. Error bars: mean ± SD

CD31, also named platelet endothelial cell adhesion molecule-1, is a crucial angiogenic marker expressed in vascular ECs [23]. Western blot analysis showed that the expression levels of CD31 in HUVECs were markedly upregulated in the SCAP-Exo-treated group compared to the non-treated control group (Fig. 2a). To detect the effects of SCAP-Exo on the vascular lumen formation in vitro, we performed a Matrigel tube formation assay and found that indexes of vascular lumen formation were significantly higher in the SCAP-Exo-treated group than in the control group as shown by an increased total tube length and increased total meshes, total branches, total node, and total junction numbers (Fig. 2b). Furthermore, we examined the effects of SCAP-Exo on blood vessel formation in vivo. We found that there were markedly more newly formed blood vessels in the subcutaneously implanted Matrigel in the SCAP-Exo group than in the control group (Fig. 2c). H&E staining also showed significantly more vascular lumen structures with more aggregated red blood cells in the SCAP-Exo-treated group than in the non-treated control group (Fig. 2d). These data suggested that SCAP-Exo improved the angiogenic capacity of ECs*.*

### SCAP-Exo mediated cell migration contributing to HUVEC angiogenesis

**Fig. 3:**
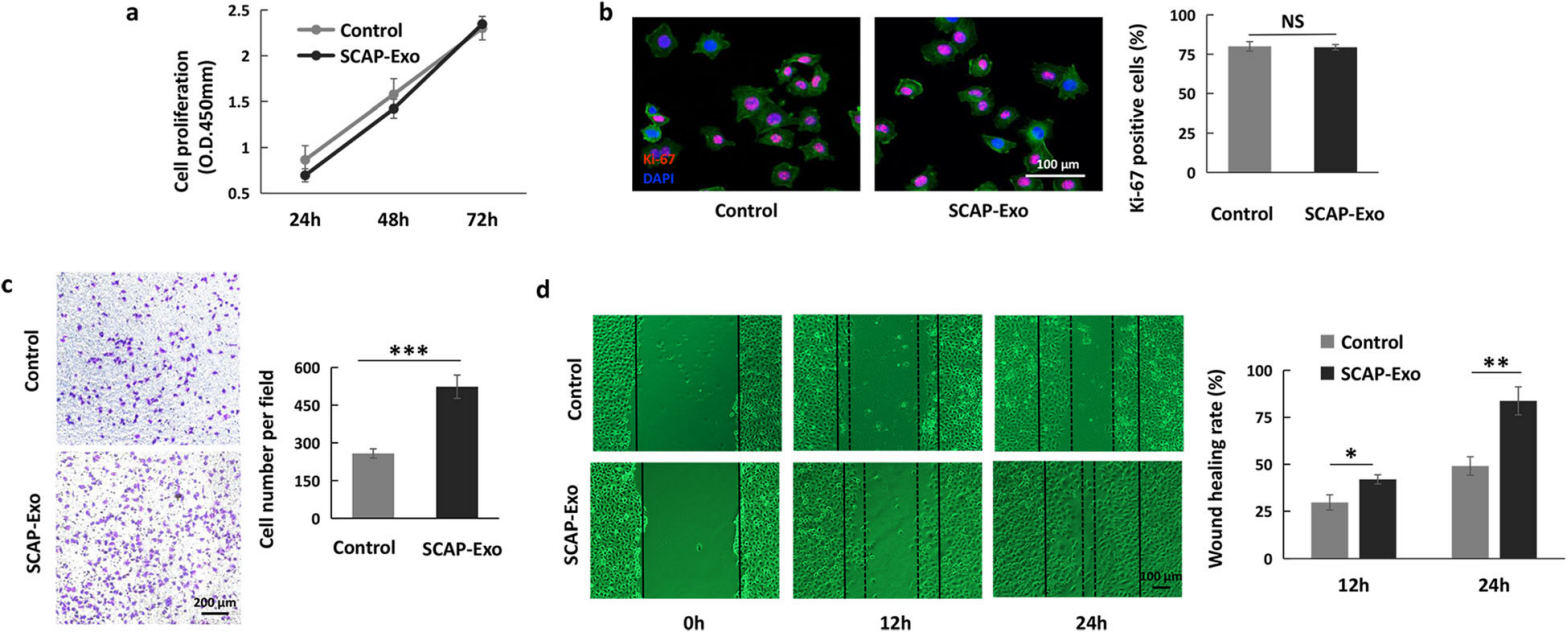
SCAP-Exo-mediated cell migration contributed to HUVEC angiogenesis. **a** A CCK-8 assay showed that SCAP-Exo treatment had no effect on the proliferation rate of HUVECs compared with the control group. **b** A Ki-67 staining assay showed that the percentage of Ki-67-positive cells (red) in the SCAP-Exo group was not different from that in the control group. The slides were counterstained with DAPI (blue) and F-actin (green). Scale bar = 100 μm. **c** A Transwell cell migration assay showed that the motility of HUVECs was upregulated in the SCAP-Exo group compared to the control group. Scale bar = 200 μm. **d** Representative images of the scratch wound healing assay showing that the wound healing rate in the SCAP-Exo group was higher at 12 h and 24 h than that in the control group. Scale bar = 100 μm. *n* = 5 in each group. NS: *P* > 0.05. *P < 0.05, **P < 0.01, ***P < 0.001. Error bars: mean ± SD

It is well known that cell migration and cell proliferation play important roles in angiogenesis of ECs [24]. To clarify how SCAP-Exo improved the angiogenic capacity of HUVECs, we first tested the proliferation of HUVECs in both the SCAP-Exo and control groups. We found that there was no significant difference in the proliferation rate of HUVECs between the SCAP-Exo group and the control group, as assessed by CCK-8 assay and Ki-67 staining (Fig. 3a, b). However, the Transwell cell migration assay showed that there were more migrated HUVECs among the SCAP-Exo-treated HUVECs than among the non-treated HUVECs (Fig. 3c). The wound healing rate of HUVECs in the SCAP-Exo group was also significantly higher at 12 and 24 h than that in the control group (Fig. 3d). These experimental data indicated that SCAP-Exo upregulated the migration of ECs to promote angiogenesis.

### SCAP-Exo increased the migration of HUVECs via Cdc42-mediated cytoskeletal reorganization

**Fig. 4:**
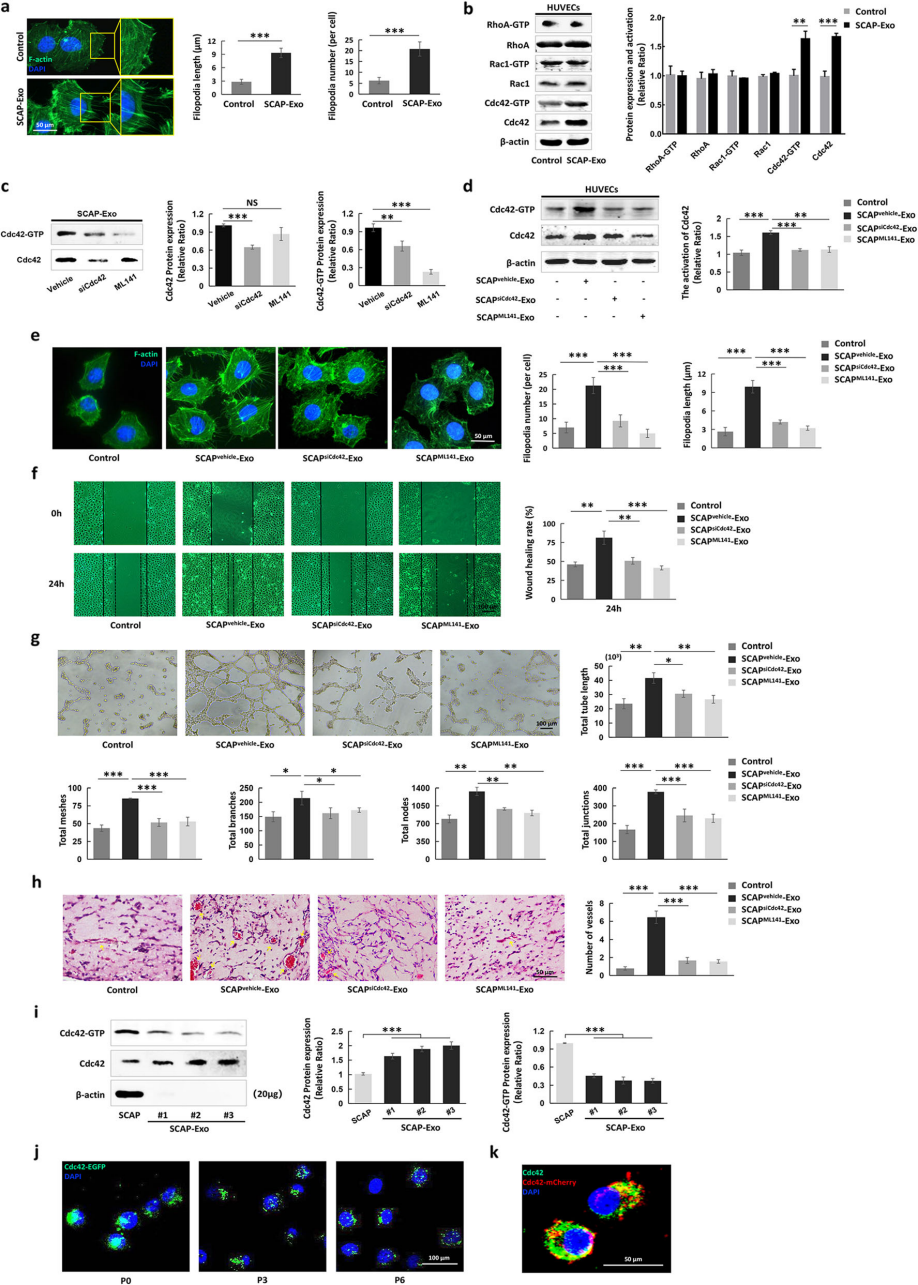
SCAP-Exo improved the migration of HUVECs via Cdc42-mediated cytoskeletal reorganization. **a** F-actin immunofluorescence staining showed that actin cytoskeleton and filopodia formation (green) was obviously greater in SCAP-Exo-treated HUVECs than in control HUVECs. The number of filopodia per cell and the filopodia length of SCAP-Exo-treated HUVECs were higher than those of control HUVECs. Scale bar = 50 μm. **b** Western blot and pull-down assays showed that the expression levels of Cdc42-GTP and Cdc42 were elevated in the SCAP-Exo-treated HUVECs, while the expression levels of RhoA and Rac1 were not significantly changed. **c** Western blot and pull-down assays showed that siCdc42 significantly reduced Cdc42 and Cdc42-GTP expression in SCAP-Exo, while ML141 mainly decreased the expression level of Cdc42-GTP in SCAP-Exo. **d** The expression levels of Cdc42 and Cdc42-GTP were significantly upregulated in the SCAP^vehicle^-Exo-treated HUVECs but not in the SCAP^siCdc42^-Exo- and SCAP^ML141^-Exo-treated HUVECs compared to the control HUVECs. **e** F-actin immunofluorescence staining showed that the actin cytoskeleton levels and the number and length of filopodia of HUVECs were higher in the SCAP^vehicle^-Exo group than in the control group but were lower in the SCAP^siCdc42^-Exo and SCAP^ML141^-Exo groups than in the SCAP^vehicle^-Exo group. Scale bar = 50 μm. **f** Representative images of the scratch wound healing assay showing that HUVEC migration in the SCAP^vehicle^-Exo group was higher than that in the control group, while the migration ability of HUVECs in the SCAP^siCdc42^-Exo and SCAP^ML141^-Exo groups was lower than that in the SCAP^vehicle^-Exo group at 24 h. Scale bar = 100 μm. **g** The in vitro tube formation assay showed that SCAP^siCdc42^-Exo- and SCAP^ML141^-Exo-treated HUVECs had a lower capacity to form vascular lumens than SCAP^vehicle^-Exo-treated HUVECs. Scale bar = 100 μm. **h** H&E staining showed that there were fewer vascular lumens (yellow arrow) containing red blood cells in the SCAP^siCdc42^-Exo and SCAP^ML141^-Exo groups than in the SCAP^vehicle^-Exo group. Scale bar = 50 μm. **i** Western blot and pull-down assays showed that SCAP-Exo had higher levels of Cdc42 expression but weaker Cdc42-GTP expression than SCAP. **j** Immunofluorescence staining showed that Cdc42**-**EGFP-labelled proteins derived from SCAP-Exo (green) were expressed steadily in the cytoplasm of HUVECs in primary culture as well as in P3 and P6 HUVECs. Scale bar = 100 μm. **k** Laser confocal microscopy image showing the co-localization of SCAP-Exo-derived Cdc42**-**mCherry (red) and Cdc42 (green) in the cytoplasm of HUVECs. The slides were counterstained with DAPI (blue). Scale bar = 50 μm. *n* = 5 in each group. **P < 0.01, ***P < 0.001. Error bars: mean ± SD

Actin cytoskeleton reorganization and pseudopodia formation provide driving forces for cell migration [25]. Accordingly, F-actin immunofluorescence staining showed that the amounts of actin cytoskeleton in the cytoplasm and the numbers of newly formed filopodia were higher in HUVECs treated with SCAP-Exo than in non-treated HUVECs, as indicated by upregulation of the number of filopodia per cell and the filopodium length (Fig. 4a). Rho GTPase family members, including RhoA, Rac1, and Cdc42, act as molecular switches to regulate cytoskeletal reorganization, contributing to cell migration [26]. Therefore, we tested the expression of Rho GTPases in both SCAP-Exo-treated HUVECs and controls. Interestingly, a pull-down assay and Western blot analysis showed that the expression levels of total Cdc42 and Cdc42-GTP were elevated in SCAP-Exo-treated HUVECs compared to non-treated HUVECs, while there were no significant differences in the expression levels of RhoA and Rac1 between groups (Fig. 4b). To determine the role of Cdc42 in SCAP-Exo-mediated EC migration, we used Cdc42 siRNA and a Cdc42 inhibitor (ML141) to downregulate Cdc42 expression in SCAP-Exo. We found that Cdc42 siRNA and the Cdc42 inhibitor (ML141) downregulated Cdc42 expression in SCAP-Exo (Fig. 4c). The total Cdc42 and Cdc42-GTP expression levels in HUVECs were not changed in SCAP^siCdc42^-Exo- or SCAP^ML141^-Exo-treated HUVECs compared with non-treated HUVECs (Fig. 4d). Moreover, we found that SCAP^siCdc42^-Exo or SCAP^ML141^-Exo induced the formation of significantly fewer and shorter filopodia in HUVECs than did SCAP^vehicle^-Exo (Fig. 4e). Moreover, the scratch wound healing assay showed that knockdown of Cdc42 in SCAP-Exo blocked the SCAP^vehicle^-Exo-mediated elevation in cell migration of HUVECs (Fig. 4f). The in vitro Matrigel tube formation assay indicated that compared to SCAP^vehicle^-Exo-treated HUVECs, SCAP^siCdc42^-Exo- and SCAP^ML141^-Exo-treated HUVECs had a reduced capacity to form vascular lumens, as shown by decreased total tube lengths and total numbers of meshes, branches, nodes, and junctions (Fig. 4g). Furthermore, H&E staining and Matrigel plug assay showed significantly decreased formation of vascular lumen structures in the SCAP^siCdc42^-Exo and SCAP^ML141^-Exo groups compared to the SCAP^vehicle^-Exo group (Fig. 4h). These data indicated that SCAP-Exo improved cytoskeletal reorganization and contributed to migration of ECs via Cdc42 signalling.

To explore the detailed mechanism of SCAP-Exo-induced Cdc42 expression in HUVECs, we used Western blot analysis to examine the protein levels of Cdc42 in SCAP and SCAP-Exo. We found that Cdc42 was steadily expressed in SCAP and SCAP-Exo derived from different SCAP populations, and Cdc42 had a higher expression level in SCAP-Exo than in SCAP (Fig. 4i). Furthermore, we transfected SCAP cells with a Cdc42-enhanced green fluorescent protein (Cdc42-EGFP) plasmid and isolated SCAP^Cdc42-EGFP^-Exo. We used SCAP^Cdc42-EGFP^-Exo to treat HUVECs and found that the Cdc42-EGFP protein derived from SCAP-Exo was transferred to HUVECs and expressed continuously in the cytoplasm of passage-0 (P0), P3, and P6 HUVECs (Fig. 4j). In addition, we produced SCAP^Cdc42-mCherry^-Exo by transfecting SCAP with a Cdc42-mCherry fluorescent protein plasmid and then used SCAP^Cdc42-mCherry^-Exo to treat HUVECs. Laser confocal microscopy showed the co-localization of Cdc42-mCherry (red) and Cdc42 (green) in the cytoplasm of SCAP-Exo-treated HUVECs (Fig. 4k). Taken together, these experimental data indicated that SCAP-Exo elevated cell migration by transferring the Cdc42 protein to improve the cytoskeletal reorganization of ECs.

### SCAP-Exo facilitated tissue regeneration of the palatal gingiva via Cdc42-mediated vascularization

**Fig. 5:**
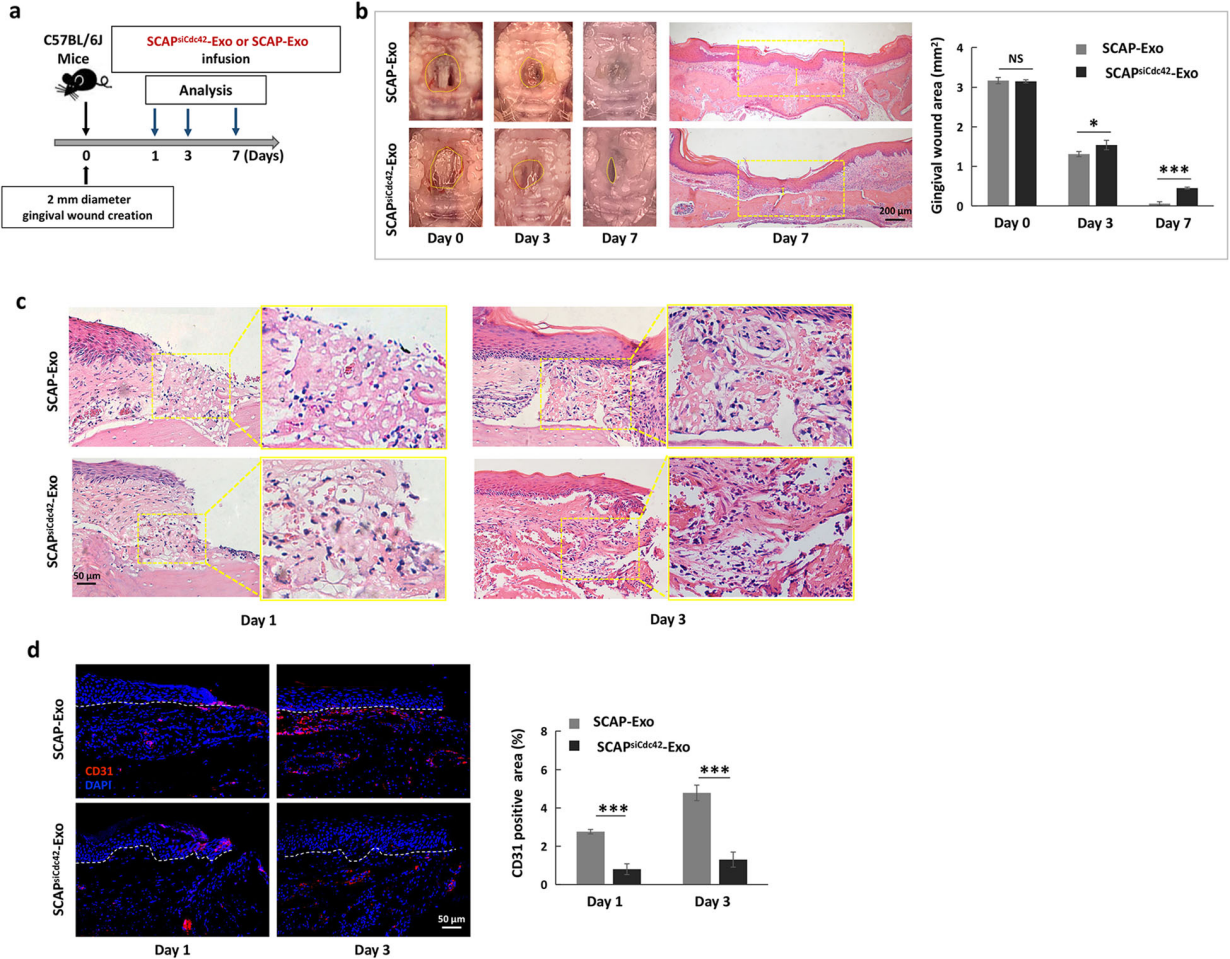
SCAP-Exo facilitated wound healing in the palatal gingiva via Cdc42. **a** Schematic indicating the experimental design for SCAP-Exo or SCAP^siCdc42^-Exo infusion in gingival wounds in the palates of mice. **b** Representative images and quantification of gingival wound areas in the SCAP-Exo and SCAP^siCdc42^-Exo groups. Wound healing of the palatal gingiva was significantly delayed in the SCAP^siCdc42^-Exo group at 3 and 7 days post wounding compared to the SCAP-Exo group. H&E staining showed that the connective tissue (yellow segment) in the SCAP^siCdc42^-Exo group was thinner than that in the SCAP-Exo group at 7 days post wounding. Scale bar = 200 μm. **c** Histological views showing fewer newly formed blood vessels containing red blood cells in the gingival wounds of the SCAP^siCdc42^-Exo group at 1 and 3 days post wounding than in the SCAP-Exo group. Scale bar = 50 μm. **d** Immunofluorescence staining and quantification showed that the percentage of CD31-positive area (red) in the SCAP^siCdc42^-Exo group was lower than that in the SCAP-Exo group at 1 and 3 days post wounding. The epidermis and connective tissue are separated by the white dotted line in the images. The slides were counterstained with DAPI (blue). Scale bar = 50 μm. *n* = 5 in each group. NS: *P* > 0.05. *P < 0.05, ***P < 0.001. Error bars: mean ± SD

We wondered whether Cdc42 derived from SCAP-Exo contributed to accelerated tissue regeneration of the palatal gingiva, so we produced Cdc42-knockdown SCAP-Exo (SCAP^siCdc42^-Exo) and infused SCAP^siCdc42^-Exo or SCAP-Exo locally into gingival wounds (Fig. 5a). We found that gingival healing was significantly delayed in the SCAP^siCdc42^-Exo group at 3 and 7 days post wounding compared to the SCAP-Exo group (Fig. 5b). H&E staining showed that in the SCAP^siCdc42^-Exo group, newly formed epidermis and a thin layer of connective tissue were present at 7 days post wounding, while in the SCAP-Exo group, the epidermal tissue was intact and continuous, and the connective tissues were markedly thickened (Fig. 5b). We further verified vascularization in the early phase of wound healing in both the SCAP^siCdc42^-Exo and SCAP-Exo groups. H&E staining showed that the SCAP^siCdc42^-Exo group exhibited fewer newly formed blood vessels than the SCAP-Exo group at 1 and 3 days post wounding (Fig. 5c). Immunostaining confirmed that knockdown of Cdc42 in SCAP-Exo blocked the SCAP-Exo-mediated upregulation of vascularization in the early phase, as indicated by the decreased percentage of CD31-positive area in the SCAP^siCdc42^-Exo group at 1 and 3 days post wounding compared to that in the SCAP-Exo group (Fig. 5d). Therefore, our data indicated that SCAP-Exo facilitated tissue regeneration of the palatal gingiva via Cdc42-mediated vascularization.

## Discussion

Angiogenesis is the formation and remodelling of new blood vessels and capillaries from outgrowth of existing blood vessels and plays a crucial role in wound healing [27]. In the process of wound healing, rapid and sufficient vascularization not only provides oxygen and nutrients to the surviving cells but also eliminates necrotic substances and controls infections [28]. Thus, the stimulation of vascularization in the early phase is the most important factor for the therapeutic effects of MSC-based tissue engineering. SCAP are the postnatal population of epidermal neural crest stem cells, which have a greater capability to promote angiogenesis than bone marrow mesenchymal stem cells (BMMSCs) [29]. In addition, neural crest-derived MSCs carry neurovascular factors such as vascular endothelial growth factor, platelet-derived growth factor, and brain-derived neurotrophic factor, which mediate the angiogenic process to improve tissue regeneration and treat ischaemic diseases [30–32]. Exosomes have cellular properties similar to those of their parent cells [33]. Here, we found that local infusion of SCAP-Exo promoted new blood vessel formation at 1 and 3 days post wounding and accelerated the healing of CSD in the palatal gingiva. Therefore, as the important paracrine factors secreted from SCAP, SCAP-Exo have excellent angiogenesis-promoting effects. Moreover, we pursued a cell-free approach, finding inspiration from the consensus that SCAP-Exo have the advantages of low immune rejection, high stability, ease of access to the wound surface, and lack of vascular obstruction [34–37]. Since the strength of wound margins is crucial for evaluating wound healing in the palatal gingiva, further study is needed to evaluate the tissue healing and functional recovery of the palatal CSD in order to fully demonstrate the effects of SCAP-Exo on soft tissue regeneration, including the strength of wound margins and the structure of collagen fibrils.

In the process of blood vessel formation, proliferating and migrating ECs interact with angiogenic factors, which results in the formation of new capillaries. Thereafter, the capillaries are covered by smooth muscle cells to form mature blood vessels with viscoelasticity and contractility [24]. Here, we found for the first time that SCAP-Exo specifically increased the cell migration of ECs, contributing to EC angiogenesis, without markedly affecting the proliferation of ECs. Cytoskeletal reorganization and pseudopodia formation play critical roles in cell migration [25]. Additionally, the formation of filopodia in SCAP-Exo-treated ECs was elevated compared to that in non-treated ECs, which confirmed the promotive effects of SCAP-Exo on cell migration. Several extracellular signals are involved in modulating the activity of microfilament-binding proteins to reorganize the cytoskeleton. The Rho GTPase family, which is composed of key downstream targets, is currently believed to promote cytoskeletal organization [26]. Cdc42, Rac1, and RhoA are the main members of the Rho GTPase family and play different roles in cytoskeletal reorganization [38]. Therefore, we examined the expression levels of Rho GTPases and found that only Cdc42 signalling was significantly upregulated in SCAP-Exo-treated ECs compared to controls. Activation of Cdc42 induces WASP-Arp2/3 complex binding by binding to the GTPase binding domain of WASP and directly regulates the polymerization of globular actin, resulting in the formation of fibrous actin (F-actin), which causes the cytoplasmic membrane to protrude outwards and form filopodia [39, 40]. Filopodia formation is the initial step of cell movement and is helpful in determining the direction of cell migration [41]. Moreover, Cdc42 is also involved in multiple mechanisms of angiogenesis. Cdc42 deletion increases ADAM17-mediated VEGFR2 shedding and reduces VEGFR2 expression on the EC surface, which indicates that Cdc42 is essential for VEGFR2-mediated signal transduction in blood vessel formation [42]. Here, we revealed a potential mechanism by which infusion with SCAP-Exo promoted the migration of ECs by activating Cdc42, leading to accelerated healing of CSD in the palatal gingiva by enhancing vascularization.

Exosomes serve as systemic cell-cell communication mediators and play an important role in MSC transplantation [43]. The underlying mechanisms are complicated but may include release of the contents to the recipient cell by endocytosis or direct combination with the molecular receptor of the recipient cell [21, 44, 45]. Here, we found that SCAP-Exo were internalized into the cytoplasm of HUVECs. We further performed co-localization and continuous passage experiments and discovered that Cdc42 derived from SCAP-Exo was directly transferred into HUVECs and reused by the HUVECs. Notably, greater Cdc42 protein enrichment was observed in SCAP-Exo than in SCAP, which suggests that SCAP-Exo might have superior performance for promotion of angiogenesis. Cdc42, a kind of Rho GTPase, continuously switches between an active GTP-binding state and an inactive GDP-binding state; the GTP can allow downstream signalling activation [46]. Interestingly, we found that not only the total Cdc42 but also the Cdc42-GTP expression levels were significantly increased in SCAP-Exo-treated ECs. In addition to total Cdc42-mediated GDP-GTP switching, guanine nucleotide exchange factors (GEFs), guanine nucleotide-activating proteins (GAPs), and guanine dissociation inhibitors (GDIs) may contribute to the active GTP-binding state of ECs [47]. The detailed mechanism by which the active GTP-binding state is induced requires further investigation.

In this study, we found, for the first time, that Cdc42 protein was expressed in MSC-derived exosomes and further revealed that exosomal Cdc42 could be transferred into ECs and reused by the recipient ECs, thus contributing to the cell migration of ECs to promote angiogenesis. Unlike the intracellular Cdc42 protein, Cdc42 in donor MSC-derived exosomes could mediate the cellular functions of recipient cells. These results indicate that MSC-derived exosomes could play a crucial role in biological crosstalk between recipient cells, which enriches the theoretical basis of MSC-derived bioactive factor-based tissue regeneration. In addition, we found that SCAP-Exo enhanced the cell migration-mediated angiogenesis of ECs, which resulted in promotion of gingival tissue regeneration in palatal CSD. Notably, gingival fibroblasts, which maintain the structural integrity of mucosal tissue, play crucial roles in the gingival regenerative process. Therefore, we cannot rule out the regulatory effects of SCAP-Exo treatment on fibroblast functions.

With the development of regenerative medicine, stem cell-based strategies have been changed to strategies based on bioactive factors. MSC-derived exosomes containing many bioactive factors could be potential experimental tools for soft tissue regeneration in the future. However, more studies are needed before SCAP-Exo can be used for angiogenesis and soft tissue regeneration in the clinic.

## Conclusion

In summary, this study revealed that local infusion of SCAP-Exo accelerated tissue regeneration of the palatal gingival CSD by promoting vascularization in the early phase. Mechanistically, SCAP-Exo improved cell migration by enhancing cytoskeletal reorganization of ECs via direct transfer of Cdc42 into the cytoplasm of recipient ECs. These findings suggest that SCAP-Exo can promote angiogenesis and provide a new strategy involving SCAP-Exo as a cell-free approach to optimize tissue regeneration in the clinic.

## Supplementary Information

**Additional file 1: Figure S1**. Characterization of SCAP. The SCAP were spindle-shaped cells in primary culture. Under in vitro osteogenic and adipogenic induction conditions, SCAP formed mineralized nodes and oil droplets, as assessed by Alizarin red S staining and oil red O staining. Flow cytometry analysis showed that SCAP expressed MSC surface markers, including CD73, CD90, and CD105, while they did not express the haematopoietic markers CD31, CD34, and CD45.

**Additional file 2: Figure S2**. Identification of SCAP-Exo. The morphology of SCAP-Exo was observed under TEM. The sizes and concentrations of SCAP-Exo were measured by nanoparticle tracking analysis. Western blot analysis showed that the exosomal surface markers Alix, CD9, and CD63 were expressed in SCAP-Exo, while calnexin was not expressed.

**Additional file 3: Figure S3**. Fate of SCAP-Exo in vivo. The in vivo tracking experiment showed that PKH-26-labelled SCAP-Exo were present in palatal gingival defects at 7 days post wounding.

**Additional file 4: Figure S4**. SCAP-Exo were endocytosed by HUVECs. Real-time live-cell imaging under a laser confocal microscope showed that an increasing number of SCAP-Exo were endocytosed by HUVECs. Analyses of the X-T axis and the Y-T axis showed the process in which SCAP-Exo were taken up by HUVECs. Three-dimensional scanning showed that SCAP-Exo existed in the cytoplasm of HUVECs.

**Additional file 5: Figure S5**. SCAP-Exo increased the expression level of the angiogenic protein CD31 in HUVECs. Western blot analysis showed that SCAP-Exo upregulated the expression levels of CD31 in HUVECs in a dose-dependent manner.

**Additional file 6: Figure S6**. Schematic diagram of SCAP-Exo-mediated promotion of the vascularization of regenerative tissue via triggering of the migration of vascular ECs. SCAP-Exo were endocytosed by ECs, and Cdc42 protein was transferred into recipient ECs to activate Cdc42/WASP/ARP2/3 cascade-mediated cytoskeletal reorganization and filopodia formation, which resulted in elevation of the cell migration of ECs and promotion of the vascularization of regenerative tissue.

## Data Availability

The data used to support the findings of this study are available from the corresponding author upon reasonable request.

## Abbreviations

CSD: Critical-size defects
MSCs: Mesenchymal stem cells
ECs: Endothelial cells
SCAP: Stem cells from apical papilla
SCAP-Exo: Exosomes derived from stem cells from apical papilla
Cdc42: Cell division cycle 42
TEM: Transmission electron microscopy
H&E: Haematoxylin and eosin
RT-LCI: Real-time live-cell imaging
HUVECs: Human umbilical vein endothelial cells
CCK-8: Cell counting kit-8
SD: Standard deviation
ANOVA: One-way analysis of variance
EGFP: Enhanced green fluorescent protein
BMMSCs: Bone marrow mesenchymal stem cells
F-actin: Fibrous actin
GEFs: Guanine nucleotide exchange factors
GAPs: Guanine nucleotide-activating proteins
GDIs: Guanine dissociation inhibitors
