# Supplementary material for: Exosomes derived from stem cells from apical papilla promote craniofacial soft tissue regeneration by enhancing Cdc42-mediated vascularization
Source: Stem Cell Res Ther. 2021 Jan 22;12:76. doi: 10.1186/s13287-021-02151-w (PMC7821694; doi:10.1186/s13287-021-02151-w)
Supplement: Supplementary file 1 — Additional file 1: Figure S1. Characterization of SCAP. The SCAP were spindle-shaped cells in primary culture. Under in vitro osteogenic and adipogenic induction conditions, SCAP formed mineralized nodes and oil droplets, as assessed by Alizarin red S staining and oil red O staining. Flow cytometry analysis showed that SCAP expressed MSC surface markers, including CD73, CD90, and CD105, while they did not express the haematopoietic markers CD31, CD34, and CD45. [file 13287_2021_2151_MOESM1_ESM.pdf]

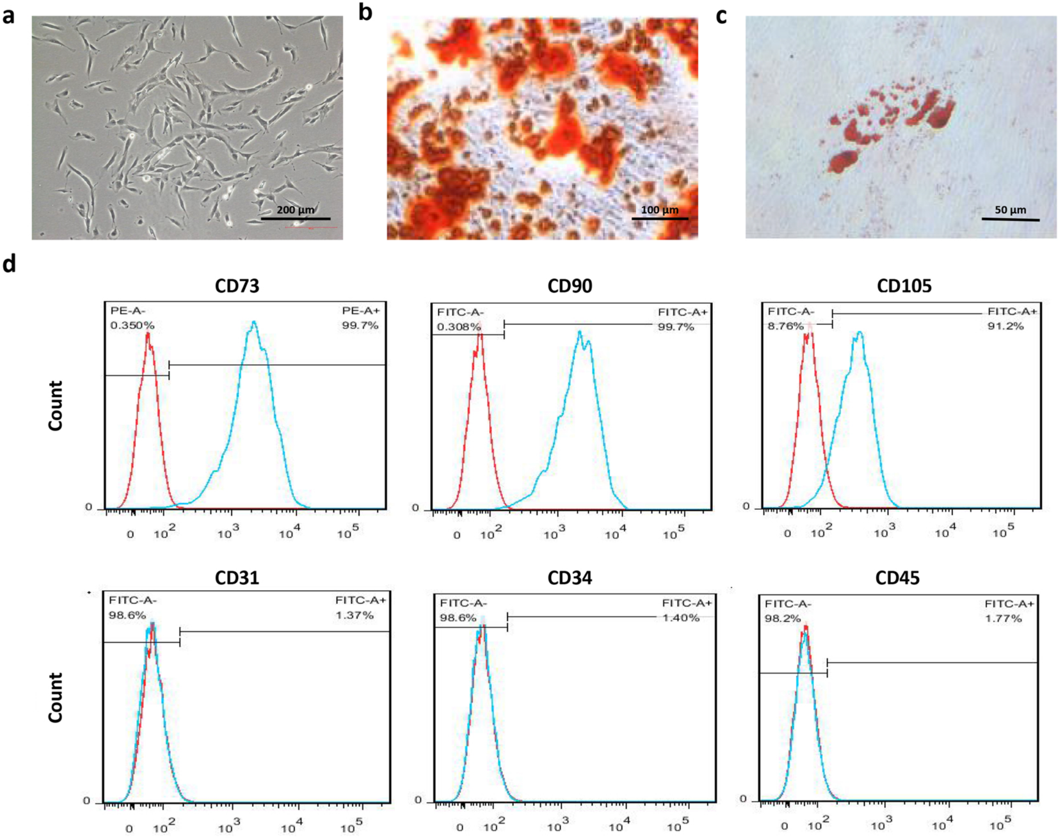

Fig. S1 Characterization of SCAP. **a** Morphology of primary SCAP. Scale bar = 200 µm. **b** Alizarin red S staining showed the formation of mineralized nodules. Scale bar = 100 µm. **c** Oil red O staining showed lipid droplet formation. Scale bar = 50 µm. **d** Flow cytometric analysis showed that SCAP were positive for CD73, CD90 and CD105 but negative for CD31, CD34 and CD45.
