# Supplementary material for: Exosomes derived from stem cells from apical papilla promote craniofacial soft tissue regeneration by enhancing Cdc42-mediated vascularization
Source: Stem Cell Res Ther. 2021 Jan 22;12:76. doi: 10.1186/s13287-021-02151-w (PMC7821694; doi:10.1186/s13287-021-02151-w)
Supplement: Supplementary file 2 — Additional file 2: Figure S2. Identification of SCAP-Exo. The morphology of SCAP-Exo was observed under TEM. The sizes and concentrations of SCAP-Exo were measured by nanoparticle tracking analysis. Western blot analysis showed that the exosomal surface markers Alix, CD9, and CD63 were expressed in SCAP-Exo, while calnexin was not expressed. [file 13287_2021_2151_MOESM2_ESM.pdf]

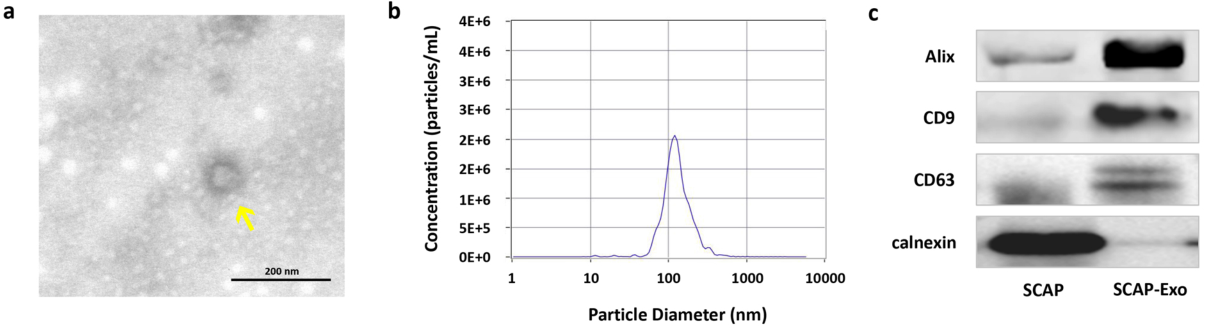

Fig. S2 Identification of SCAP-Exo. **a** The morphology of SCAP-Exo was observed under TEM. The yellow arrow indicates an exosome. Scale bar = 200 nm. **b** The sizes and concentrations of SCAP-Exo were measured by nanoparticle tracking analysis. **c** Western blot analysis showed that the exosomal surface markers Alix, CD9, and CD63 were expressed in SCAP-Exo, while calnexin was not expressed.
