# Supplementary material for: Exosomes derived from stem cells from apical papilla promote craniofacial soft tissue regeneration by enhancing Cdc42-mediated vascularization
Source: Stem Cell Res Ther. 2021 Jan 22;12:76. doi: 10.1186/s13287-021-02151-w (PMC7821694; doi:10.1186/s13287-021-02151-w)
Supplement: Supplementary file 3 — Additional file 3: Figure S3. Fate of SCAP-Exo in vivo. The in vivo tracking experiment showed that PKH-26-labelled SCAP-Exo were present in palatal gingival defects at 7 days post wounding. [file 13287_2021_2151_MOESM3_ESM.pdf]

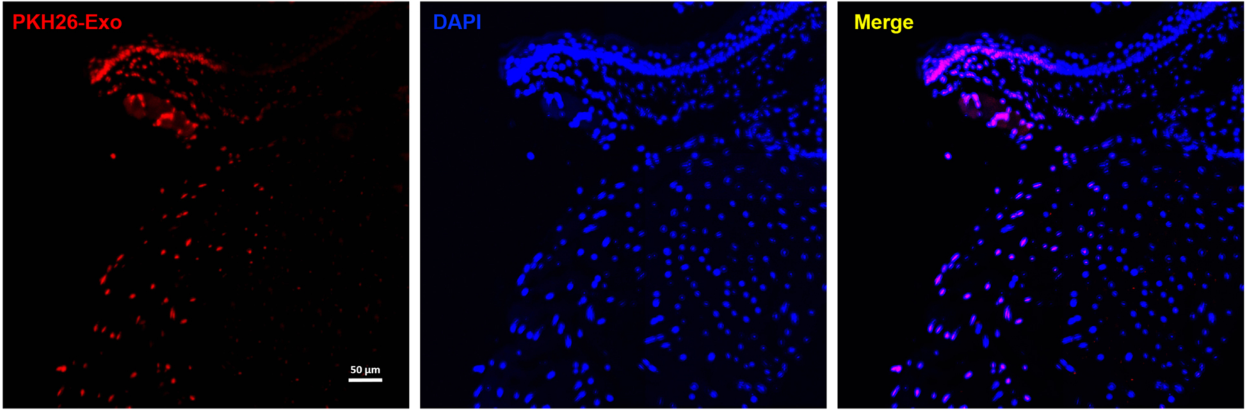

Fig. S3 Fate of SCAP-Exo in vivo. The in vivo tracking experiment showed that PKH-26-labelled SCAP-Exo were present in palatal gingival defects at 7 day post wounding. Scale bar = 50 μm.
