# Supplementary material for: Exosomes derived from stem cells from apical papilla promote craniofacial soft tissue regeneration by enhancing Cdc42-mediated vascularization
Source: Stem Cell Res Ther. 2021 Jan 22;12:76. doi: 10.1186/s13287-021-02151-w (PMC7821694; doi:10.1186/s13287-021-02151-w)
Supplement: Supplementary file 4 — Additional file 4: Figure S4. SCAP-Exo were endocytosed by HUVECs. Real-time live-cell imaging under a laser confocal microscope showed that an increasing number of SCAP-Exo were endocytosed by HUVECs. Analyses of the X-T axis and the Y-T axis showed the process in which SCAP-Exo were taken up by HUVECs. Three-dimensional scanning showed that SCAP-Exo existed in the cytoplasm of HUVECs. [file 13287_2021_2151_MOESM4_ESM.pdf]

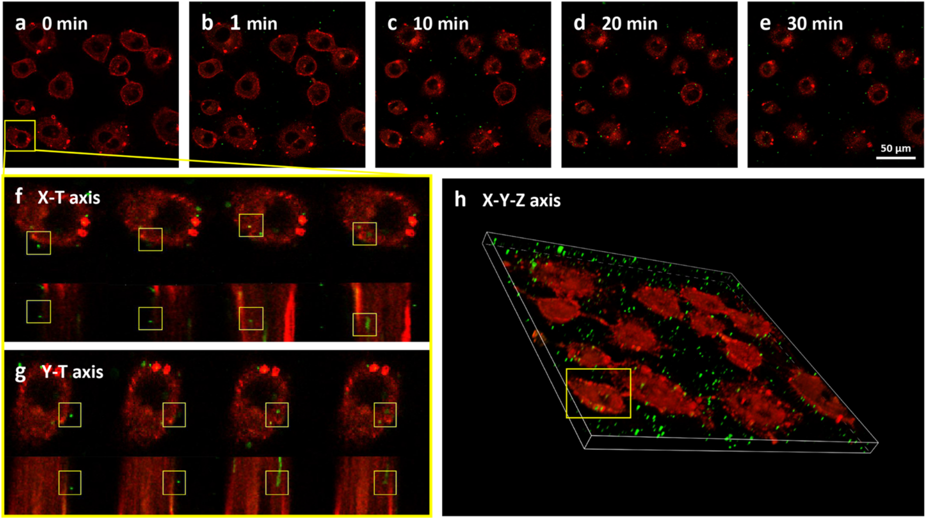

Fig. S4 SCAP-Exo were endocytosed by HUVECs. **a-e** Real-time live-cell imaging under a laser confocal microscope showed that an increasing number of SCAP-Exo were endocytosed by HUVECs. Scale bar = 50 μm. **f-g** Analyses of the X-T axis and Y-T axis showed the process by which SCAP-Exo were taken up by HUVECs. **h** Three-dimensional scanning showed that SCAP-Exo existed in the cytoplasm of HUVECs.
