# Supplementary material for: Exosomes derived from stem cells from apical papilla promote craniofacial soft tissue regeneration by enhancing Cdc42-mediated vascularization
Source: Stem Cell Res Ther. 2021 Jan 22;12:76. doi: 10.1186/s13287-021-02151-w (PMC7821694; doi:10.1186/s13287-021-02151-w)
Supplement: Supplementary file 5 — Additional file 5: Figure S5. SCAP-Exo increased the expression level of the angiogenic protein CD31 in HUVECs. Western blot analysis showed that SCAP-Exo upregulated the expression levels of CD31 in HUVECs in a dose-dependent manner. [file 13287_2021_2151_MOESM5_ESM.pdf]

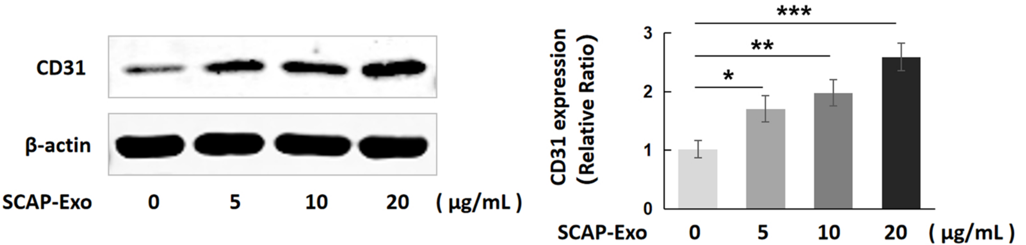

Fig. S5 SCAP-Exo increased the expression level of the angiogenic protein CD31 in HUVECs. Western blot analysis showed that SCAP-Exo upregulated the expression levels of CD31 in HUVECs in a dose-dependent manner.
