# Supplementary material for: Exosomes derived from stem cells from apical papilla promote craniofacial soft tissue regeneration by enhancing Cdc42-mediated vascularization
Source: Stem Cell Res Ther. 2021 Jan 22;12:76. doi: 10.1186/s13287-021-02151-w (PMC7821694; doi:10.1186/s13287-021-02151-w)
Supplement: Supplementary file 6 — Additional file 6: Figure S6. Schematic diagram of SCAP-Exo-mediated promotion of the vascularization of regenerative tissue via triggering of the migration of vascular ECs. SCAP-Exo were endocytosed by ECs, and Cdc42 protein was transferred into recipient ECs to activate Cdc42/WASP/ARP2/3 cascade-mediated cytoskeletal reorganization and filopodia formation, which resulted in elevation of the cell migration of ECs and promotion of the vascularization of regenerative tissue. [file 13287_2021_2151_MOESM6_ESM.pdf]

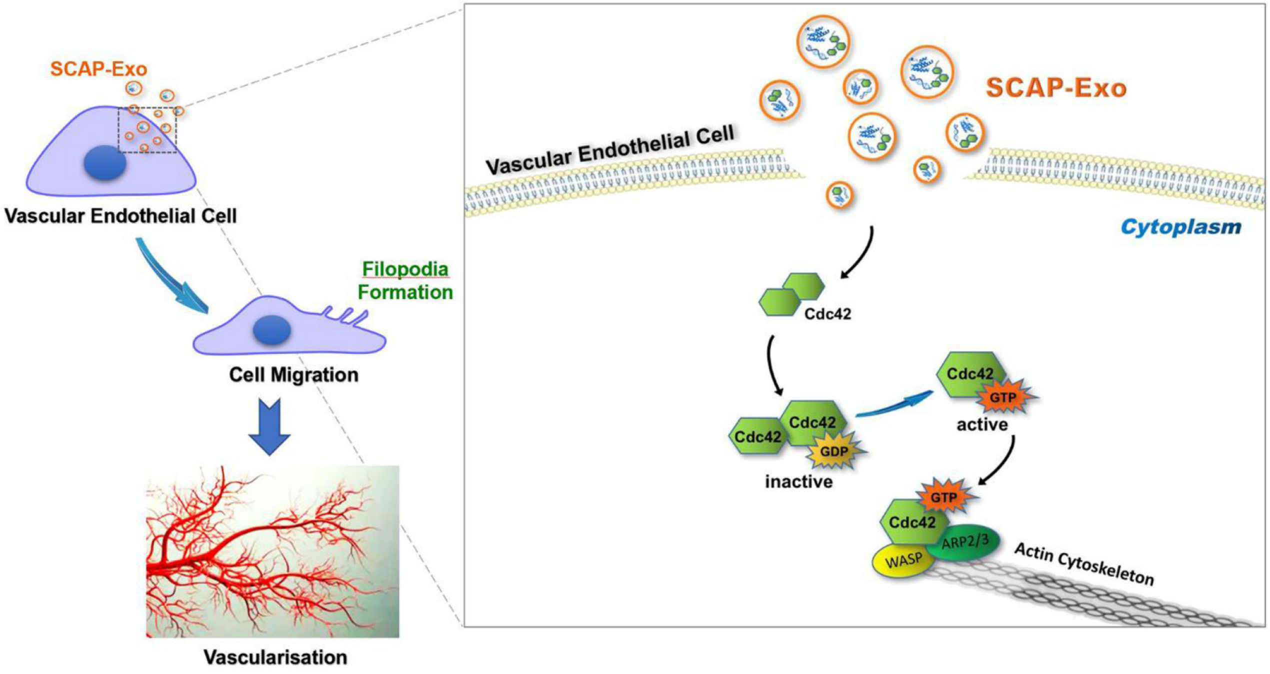


**Fig. S6** Schematic diagram of SCAP-Exo-mediated promotion of the vascularization of regenerative tissue via triggering of the migration of vascular ECs. SCAP-Exo were endocytosed by ECs, and Cdc42 protein was transferred into recipient ECs to activate Cdc42/WASP/ARP2/3 cascade-mediated cytoskeletal reorganization and filopodia formation, which resulted in elevation of the cell migration of ECs and promotion of the vascularization of regenerative tissue.
